# Short-Term Exposure to PM_2.5_ Chemical Components and Depression Outpatient Visits: A Case-Crossover Analysis in Three Chinese Cities

**DOI:** 10.3390/toxics12020136

**Published:** 2024-02-07

**Authors:** Zitong Zhuang, Dan Li, Shiyu Zhang, Zhaoyang Hu, Wenfeng Deng, Hualiang Lin

**Affiliations:** 1 School of Public Health, Sun Yat-Sen University, No. 74 Zhongshan Road 2, Guangzhou 510080, China; 2 Huizhou Center for Disease Control and Prevention, No. 10 Jiangbei Fumin Road, Huizhou 516003, China; dengwf6@outlook.com

**Keywords:** PM_2.5_ chemical components, depression, time-stratified case-crossover analysis, distributed lag nonlinear model

## Abstract

Background: The association between specific chemical components of PM_2.5_ and depression remains largely unknown. Methods: We conducted a time-stratified case-crossover analysis with a distributed lag nonlinear model (DLNM) to evaluate the relationship of PM_2.5_ and its chemical components, including black carbon (BC), organic matter (OM), sulfate (SO_4_^2−^), nitrate (NO_3_^−^), and ammonium (NH_4_^+^), with the depression incidence. Daily depression outpatients were enrolled from Huizhou, Shenzhen, and Zhaoqing. Results: Among 247,281 outpatients, we found the strongest cumulative effects of PM_2.5_ and its chemical components with the odd ratios (ORs) of 1.607 (95% CI: 1.321, 1.956) and 1.417 (95% CI: 1.245, 1.612) at the 50th percentile of PM_2.5_ and OM at lag 21, respectively. Furthermore, the ORs with SO_4_^2−^ and NH_4_^+^ at the 75th percentile on the same lag day were 1.418 (95% CI: 1.247, 1.613) and 1.025 (95% CI: 1.009, 1.140). Relatively stronger associations were observed among females and the elderly. Conclusions: Our study suggests that PM_2.5_ and its chemical components might be important risk factors for depression. Reducing PM_2.5_ emissions, with a particular focus on the major sources of SO_4_^2−^ and OM, might potentially alleviate the burden of depression in South China.

## 1. Introduction

Depression is one of the world’s most prevalent mental diseases, marked by abnormal mood swings and transient emotional responses to the challenges of daily life [1]. According to the World Health Organization (WHO), depression affects approximately 3.8% of the global population, especially in developing countries [1]. In China, depression has been estimated to have a prevalence of 3.6% [2], making it the second leading cause of years lived with disability [3]. Exploring the risk factors for depression is of great public health significance for prevention as well as the reduction of the burden on society.

It has been demonstrated that a few risk factors, including genetic factors [4], parental depression and stressful life events [5], other psychological disorders [5], and socioeconomic factors [6], could trigger the onset of depression. Moreover, there is increasing evidence supporting the notion that ambient air pollution has a significant impact on the risk of depression. Particularly, it is widely recognized that ambient fine particulate matter (PM_2.5_) pollution exposure is associated with depression. For example, one cohort study from UK Biobank has shown that each interquartile range (IQR) increase in PM_2.5_ was associated with a hazard ratio (HR) of 1.08 (95% CI: 1.07, 1.10) in depression [7]. One study in Ningbo indicated that the essential effects of PM_2.5_ on depression were found with an excess risk (ER) of 2.59 (95% CI: 0.72, 4.49) on lag0 [8]. In addition, Tsai SS et al. reported that an IQR increase in the PM_2.5_ concentration leads to a 17% (95% CI: 14%, 19%) rise in the risk of depression [9]. Additionally, a meta-analysis including 39 studies supported a significant association between short-term ambient PM_2.5_ exposure and the risk of depression, with a relative ratio (RR) of 1.009 (95% CI: 1.007, 1.011) for each 10 µg/m^3^ increase in PM_2.5_ [10].

However, previous studies have proposed that the associations between PM_2.5_ and depression vary across different populations and areas, which might be due to differences in the harmful components of PM_2.5_ [11,12]_,_ including black carbon (BC), organic matter (OM), sulfate (SO_4_^2−^), nitrate (NO_3_^−^), and ammonium (NH_4_^+^). For example, each 1 unit increase in the BC and OM concentrations was associated with depressive symptoms and the relative risk was 1.118 (95% CI: 1.020, 1.225) and 1.134 (95% CI: 1.028, 1.252), respectively [11]. However, it is still unknown whether short-term specific PM_2.5_ chemical component exposure contributes to the risk of depression. 

Given these research gaps, we conducted a time-stratified case-crossover analysis with a distributed lag nonlinear model (DLNM) in three subtropical cities in South China. We aimed to identify the most toxic components responsible for the nonlinear and delayed effects of PM_2.5_ and its chemical components on outpatient visits for depression. 

## 2. Material and Methods

### 2.1. Study Area

The study area covered three subtropical cities in Guangdong Province: Huizhou, Shenzhen, and Zhaoqing. These three cities are located in the southeast coastal area of China, covering an area of 5.72 million km^2^, 15.87 million km^2^, and 4.04 million km^2^, respectively. According to the seventh national population census, the permanent populations of Huizhou, Shenzhen, and Zhaoqing were 6.04 million, 17.56 million, and 4.11 million, respectively [13]. 

### 2.2. Outcome

Daily outpatient visits for depression were collected at psychiatric specialist hospitals in each study city (the Second People’s Hospital in Huizhou from 1 September 2013 to 11 November 2018, Kangning Hospital in Shenzhen from 1 August 2016 to 31 December 2018, and the Third People’s Hospital in Zhaoqing from 1 August 2016 to 12 January 2018). Personal information, consisting of age, gender, visit date for depression, clinical diagnosis, and residential address, was extracted from the electronic medical record systems. Only patients who have lived in the cities for more than 6 months and have complete information were included in the analysis. Depression was identified by the International Classification of Disease-10 (ICD-10) codes F32–F33. 

### 2.3. Air Pollution Exposure Assessment

According to previous studies [11,12], our investigation focused on black carbon (BC), organic matter (OM), sulfate (SO_4_^2−^), nitrate (NO_3_^−^), and ammonium (NH_4_^+^), as they are the primary chemical components of PM_2.5_, all of which are closely associated with depression. Exposure assessments of PM_2.5_ and these components were conducted based on the China Tracking Air Pollution (TAP, http://tapdata.org.cn/, accessed on 30 December 2023), as calculated with a spatial resolution of 10 km × 10 km. It collected data from a variety of sources, including ground observations, satellite aerosol optical depth (AOD), and others. After data collection and processing, a Two-Stage Machine Learning Model and Gap-Filling Method were approached to model the PM_2.5_ [14]. Detailed information can be found in the study by Geng G et al. The average out-of-bag cross-validation correlation coefficient (R) for the PM_2.5_ quality in the TAP data source was 0.72, and the model cross-validation Rs were over 0.67 for the individual components, representing an overall good performance on the ground estimation of the PM chemical components [14,15]. 

Meteorological data, including the ambient temperature (°C) and dew point temperature (°C), were derived from the open-access ERA-5 reanalysis of the European Center for Medium-Range Weather Forecasts (ECMWF) (spatial resolution, 9 km × 9 km) [16]. The ECMWF has one of the largest supercomputer facilities and meteorological data archives in the world and produces global numerical weather forecasts for users worldwide. On the basis of the ambient temperature and dew point temperature, we determined the relative humidity for further analysis. 

Based on the residential address geocoded into the latitude and longitude data, we used a bilinear interpolation method to estimate the daily PM_2.5_ and its chemical components exposure, as well as the meteorological variables, for each outpatient. The weighted average of the four closest grids was used to calculate the final concentration, and we averaged the exposure for all the outpatients from the three cities as the individual-level exposure.

### 2.4. Statistical Analysis

The mean and standard deviation (SD) were used to describe the continuous variables, while the number and percentage were employed to describe the categorical variables in our study. The Spearman’s correlation was determined to examine the relationship between PM_2.5_ and its chemical components as well as meteorological factors. 

A time-stratified case-crossover design was employed in our study, using the case day as the hospital visiting day and designating days of the week that shared the same year, month, and week as the control days. For example, if a patient presented at the hospital with depression on Friday, 1 March 2017, that date was designated as the case day, while all the other Fridays in March 2017 (8, 15, and 22 March) were designated as the control days. This design could control for any time-invariance at the patient level (such as age, gender, and genetic) [17], which helps to avoid the influence of uncontrollable factors and increase comparability when reducing bias by taking itself as the control. 

Because of the nonlinear and lagged relationship between air pollution exposure and health-related outcomes supported in previous studies [18,19], we adopted a DLNM approach that incorporates cross-basis functions. This allowed us to simultaneously express the nonlinear exposure–response and lag–response associations [20]. Conditional logistic models were developed to investigate the individual-level associations between the risk of depression outpatient visits and short-term exposures to ambient air pollutants [21].

To account for the short-term exposure patterns in depression patients and other time-varying factors that may obfuscate the connection between air pollution (PM_2.5_ and its chemical components) and depression outpatient visits, the *df* for both the air pollution and lag structure in the model was set to 4 after detailed considerations of both the relevant literature [22] and the Akaike information criterion (AIC) (Table S1). In line with previous studies, the daily temperature and relative humidity were both modeled using natural cubic splines with 4 *df* [23]. Based on the Diagnostic and Statistical Manual of Mental Disorders-5th Edition (DSM-5) requirement that the symptoms associated with depressive disorders must last for at least two weeks before being diagnosed officially as depression, we considered 21 as the maximum number of lag days and the lag effect was set as single-day lags. After the main model was established, we included exposure data in the model as a daily average. The association of different PM_2.5_ chemical component exposures with the depression incidence was calculated respectively, so as to explore the susceptible windows. The concentrations with minimum effects of air pollutants were taken as a reference for estimating the odd ratios (ORs).

This study categorized the outpatients by gender (male or female) and age (under or over 60 years old) to identify potentially susceptible subgroups.

In the sensitivity analysis, we changed separately the *df* of 3 and 5 for the daily temperature and relative humidity. R software (version 4.2.2) was employed for all the analyses. The statistical tests were two-sided, and a relationship was judged statistically significant when *p* 0.05 was obtained. 

## 3. Results

### 3.1. Descriptive Results

A total of 58,112 outpatients in Huizhou, 189,156 in Shenzhen, and 27,013 in Zhaoqing were recorded with depression in our study (Table S2). Almost half of the visitors were females (56.83%), and the majority (89.43%) were under 60 years old (Table 1). The mean concentration of PM_2.5_, BC, OM, SO_4_^2−^, NO_3_^−^, and NH_4_^+^ on the case day was 29.03 μg/m^3^, 1.71 μg/m^3^, 8.11 μg/m^3^, 6.00 μg/m^3^, 3.86 μg/m^3^, and 3.00 μg/m^3^, respectively (Table 2). Figure S1 showed that the exposure levels for more than half of the study population have not yet reached the average concentrations of PM_2.5_ and its components (on the case day). The daily exposure to PM_2.5_ and its chemical components ranged across the different cities (Table S2). For example, the mean exposure to NO_3_^−^ in Zhaoqing was 5.50 μg/m^3^, marginally higher than that in Shenzhen (3.42 μg/m^3^) and Huizhou (4.52 μg/m^3^). 

The study found that the correlations between PM_2.5_ and its chemical components are ranging from moderate to strong, with a correlation coefficient (r) higher than 0.50 (Table S3). For instance, BC showed a strong correlation with PM_2.5_ (r = 0.93) and a moderate correlation with NO_3_^−^ (r = 0.76). By contrast, the daily temperature and relative humidity were negatively associated with the PM_2.5_ chemical components.

### 3.2. The Associations of PM_2.5_ and Its Chemical Components with Outpatient Visits for Depression

The overall exposure–response relationships of short-term PM_2.5_ and its chemical components exposure with outpatient visits for depression at lag 21 are shown in Figure S2. Inverted S-shape curves were observed for depression, which clearly demonstrated that the associations of PM_2.5_ and its chemical components with depression outpatient visits are nonlinear. Specifically, the risks associated with PM_2.5_ and its chemical components increased steadily as the concentrations increased from a low level, with turning points at approximately 25.9 μg/m^3^ for PM_2.5_ and 1.3 μg/m^3^ for BC, 7.2 μg/m^3^ for OM, 4.0 μg/m^3^ for SO_4_^2−^, 3.3 μg/m^3^ for NO_3_^−^, and 2.7 μg/m^3^ for NH_4_^+^, after which the risks marginally decreased with the increase in concentrations. PM_2.5_ demonstrated the strongest association with outpatient visits for depression. When the level of the air pollution reached especially high, the risks increased sharply. Moreover, the concentration range corresponding to the peak number of outpatients aligned with that of the turning points (Figure S1).

In the three cities, consistent, nonlinear effects of PM_2.5_ and its chemical components were observed on depression outpatient visits (Figure S3). In Huizhou, the components analyzed in this study reached their own turning points at relatively later concentrations. Except for NO_3_^−^, the risks of PM_2.5_ and the other four chemical components increased most sharply at high exposure concentrations in Zhaoqing.

Figure S4 shows the relationships of short-term exposure to PM_2.5_ and its chemical components with the morbidity of mental disorders at lag 14. Compared to the curves shown in Figure S2, the risks were marginally decreased for outpatient visits for depression at the same level of exposure to PM_2.5_ and its chemical components, particularly at both low and high concentrations.

**Figure 1:**
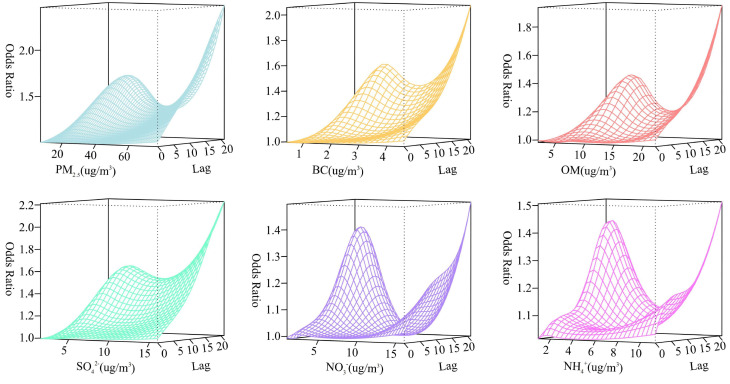
3D plot of the odd ratios (OR) among the PM_2.5_ chemical components and lag days for the outpatient visits for depression using a DLNM method.

Figure 1 shows the specific effects of PM_2.5_ and its chemical components exposure on depression outpatient visits, taking into account different concentrations and lag days. The curves show a U-shape or an inverted S-shape, with a relatively higher risk observed at high concentration exposure. Thus, the concentrations were divided into the lower percentile (25th percentile), median (50th percentile), and upper percentile (75th percentile) for further investigation to determine their effects. Additionally, it was found that as the lag time increased, PM_2.5_ and its chemical components exposure had a significant impact on the risk of depression outpatient visits. 

Table 3 shows the cumulative risks of PM_2.5_ and its chemical components for depressive outpatients. The cumulative risks of PM_2.5_ continued to increase as the lag days extended, with the maximum OR of 1.525 (95% CI: 1.231, 1.889) (25th percentile), 1.607 (95% CI: 1.321, 1.956) (50th percentile), and 1.403 (95% CI: 1.160, 1.697) (75th percentile) at a 21–day lag, respectively. Significant associations were consistently found for SO_4_^2−^ and NH_4_^+^, and increased to the maximum at 7.8 μg/m^3^ (ORs = 1.418, 95% CI: 1.247, 1.613) and 4.7 μg/m^3^ (OR = 1.086, 95% CI = 1.054, 1.120), respectively. OM was significantly associated with depression outpatient visits, and the highest cumulative risks reached 1.417 (95% CI: 1.245, 1.612) at 7.2 μg/m^3^ (50th percentile) at lag 21 days. Conversely, for BC and NO_3_^−^, we have not observed appreciable cumulative risk associations with depression outpatient visits.

PM_2.5_ and its chemical components appeared to be strongly associated with outpatient visits for depression across the three cities (Table S4), with the lag patterns similar to those observed in all the study populations. For example, for a 26.0 μg/m^3^ increase in PM_2.5_ at lag21, the OR of the outpatient visits for depression was 1.465 (95% CI: 1.091, 1.967) in Shenzhen. Significant associations were observed for SO_4_^2−^ and OM. For instance, the maximum ORs of SO_4_^2−^ and depression outpatient visits in Huizhou, Shenzhen, and Zhaoqing was 1.359 (95% CI: 1.148, 1.610), 1.146 (95% CI: 0.998, 1.317) and 1.476 (95% CI: 1.055, 2.066), respectively. Additionally, no significant results were observed for BC, NO_3_^−^, and NH_4_^+^ in all three cities.

### 3.3. Associations by Gender and Age

Figures S5 and S6 show that PM_2.5_ and its chemical components had different effects on depression outpatient visits in the gender and age analyses. More pronounced associations were observed for PM_2.5_, OM, and SO_4_^2−^ in females, with corresponding ORs of 1.820 (95% CI: 1.398, 2.369), 1.561 (95% CI: 1.314, 1.855), and 1.380 (95% CI: 1.206, 1.578) at lag 21 days (Table S5). The elderly (over 60 years old) were marginally more vulnerable to PM_2.5_ and OM than the young (under 60 years) based on age analysis (Table S6).

### 3.4. Sensitivity Analyses

Sensitivity analyses demonstrated that when we change the *df* for the daily temperature (*df* = 3 or 5) and relative humidity (*df* = 3 or 5), the associations remained almost similar (Figures S7 and S8, Tables S7 and S8). While for NO_3_^−^, the *df* for the daily temperature and relative humidity both changed to 3, the corresponding ORs for NO_3_^−^ were 1.045 (95% CI: 1.027, 1.063) at lag 7 days, 1.123 (95% CI: 1.095, 1.151) at lag 14 days, 1.148 (95% CI: 1.112, 1.185) at lag 21 days. The robustness of our study was demonstrated by the fact that the additional analytical results were largely compatible with the main model.

## 4. Discussion

This study is the first to investigate the association between short-term exposure to five major chemical components of PM_2.5_ and outpatient visits for depression. Using a time-stratified case-crossover study with DLNM in three subtropical cities in Guangdong Province, Huizhou, Shenzhen, and Zhaoqing, we revealed that short-term exposure to PM_2.5_, BC, OM, SO_4_^2−^, NO_3_^−^, and NH_4_^+^ elevated the risk of outpatient visits for depression. The association between SO_4_^2−^ and depression appeared to be more consistent across different lag days. OM has been shown to be the strongest associated with depression outpatient visits when exposed at the median concentration. Stratified analyses yielded pronounced results in females and the elderly.

Consistent with our findings, a comprehensive systematic review found an elevated risk of depression related to short-term PM_2.5_ exposure [10]. Another study, conducted in nine cities in the Beijing–Tianjin–Hebei area, indicated that PM_2.5_ exposure corresponded to a 1.92 (95% CI: 1.19, 3.12) rise in depression visits [24]. Hong J et al. highlighted that as the level of air pollution increased, the overall risks of depression, as well as other mental disorders, responded with nonlinear curves, which was in line with an inverted S-shape curve observed in our study [18]. Given the slight reduction after the turning points in the curves, it is believed that individual behavioral modifications (staying indoors, limiting physical activity) partially mitigate the effect of air pollution on the onset of depression [25]. From the standpoint of the biological mechanism, this might be related to disease competition and saturation of biochemical sites, such as receptor competition and enzyme activity [26]. We indeed advocate taking necessary measures to protect ourselves when there is a high level of air pollution. The delayed effect differs from the findings of a multi-city study including 111,842 hospital outpatient visits in China, which reported an excess relative risk (EER) (%) of 1.039% (95% CI: 1.344, 1.739%) associated with a 10 μg/m^3^ increase at lag 05 of PM_2.5_ [27]. 

The association found between the concentrations of PM_2.5_ and depression outpatient visits varied across the three cities studied, which was attributed to the regional specificity of the chemical components of PM_2.5_ [11], genetic factors [28], parental depression and stressful life events [5], and socioeconomic factors [6]. In terms of the multiple major components of PM_2.5_, our study was strongly corroborated by Ju K et al., who discovered evidence of a positive correlation between long-term exposure to PM_2.5_ chemical components and depression [11]. 

It was biologically plausible that PM_2.5_ might trigger the onset of depression. Existing research reported that PM_2.5_ was related to oxidative stress, inflammatory responses [29], and neurotransmitter imbalances such as serotonin and norepinephrine [30], which were associated with depression. Moreover, the aforementioned toxic effects might account for the deposition of special PM_2.5_ chemical components. In addition, several other studies revealed that the association of PM_2.5_ with depression was not significant [31]. Therefore, identifying the association of both PM_2.5_ and its chemical components with depression is necessary.

As a significant component of PM_2.5_, BC primarily forms from incomplete combustion of fossil fuel [32]. In agreement with our findings, Shen M et al. demonstrated that exposure to BC was substantially associated with depression (*β* = 0.17, *p* < 0.001) in college students [12]. Combining the results of the overall risk in Figure 1 and the cumulative risk in Table 3, we found that the cumulative effect is reversed at longer lags, and the same situation occurs again for NO_3_^−^. Different from BC, OM is released into the environment both by combustion emissions and photochemical reactions [33]. On the other hand, the compounds of OM are complex, accounting for organic carbon, polycyclic aromatic hydrocarbons (PAHs), zero phthalates, etc. The associations between OM and depression outpatient visits were explored in our study, supported by a previous study in which the OR of an increase of 1 unit was 1.134 (95% CI: 1.028, 1.252) [11]. Interestingly, the median concentrations of OM corresponded to the first turning point concentration in Figure S2, which may explain the higher cumulative risks of these two pollutants observed at 7.2 μg/m^3^ (50th percentile) in Table 3. In terms of the mechanism, when OM cooperates with the other PM_2.5_ components that are inhaled and exert toxic effects, there is a certain competitive relationship in the pathways [11]. PAHs are considered to be both the precursors of BC [34] and the important components of OM [35]. Rahman et al. reported that the concentration levels of seven types of PAHs in urine were positively correlated with depression [36]. Mechanisms studies supported the notion that BC might lead to oxidative stress and inflammatory injury along with PAHs, which may contribute to neurotoxicity [34]. The exact mechanisms of the association between OM and depression have been largely unclear. Thus, more investigation is warranted to explore the mechanisms underlying the associations between OM and depression.

NH_4_^+^ is a secondary inorganic aerosol present in the air, primarily in the form of a mixture of nitrate and sulfate [37]. In China, industrial production, agricultural activities, and transportation emissions are considered to be the major sources of NH_4_^+^ [37]. We found that short-term NH_4_^+^ exposure was associated with the risk of daily depression outpatient visits, especially in Huizhou, which revealed an OR of 1.141 (95% CI: 1.022, 1.273). NO_3_^−^ and SO_4_^2−^ are the other two secondary inorganic aerosols formed in the atmosphere. NO_3_^−^ is generated mainly from the photochemical conversion of nitric acid and ammonia [38], while SO_4_^2−^ is usually emitted from the combustion of fossil fuels [35]. Our study also discovered that NO_3_^−^ and SO_4_^2−^ were significantly associated with depressive symptoms, whereas stronger associations were observed for SO_4_^2−^. A cohort study of Chinese adults has pointed out that long-term exposure to these three inorganic components elevated the incidence of depression, with the ORs found being 1.127 (95% CI: 1.011, 1.255) for NH_4_^+^, 1.117 (95% CI: 1.020, 1.224) for SO_4_^2−^, and 1.107 (95% CI: 0.981, 1.248) for NO_3_^−^ [11]. Unfortunately, the reasons for these findings have not been fully established. Experimental studies proposed that NH_4_^+^ may induce damage to glial cells and block the maturation of neurons [39], while NO_3_^−^ and SO_4_^2−^ can cause mitochondrial abnormalities [40]. 

Our stratified analyses revealed that PM_2.5_ was more strongly associated with depression outpatient visits in females, which was in line with previous studies [8]. A study including 26 Chinese cities suggested that an IQR increase in PM_2.5_ concentrations corresponding to a 3.97% (95% CI: 2.06, 5.91) increase in admissions for females, while males experienced a minor increase of 0.74% (95% CI: 1.92, 3.47) [19]. Neurodevelopment and hormone states [41] seem to be significant explanations for the difference, but more research is warranted. This study found that the elderly were more susceptible to PM_2.5_ and its chemical components, which was aligned with previous research. Wang et al. discovered that PM_2.5_ exposure had an important influence on hospitalization for depression among individuals over the age of 65, with an OR of 9.23 (95% CI: 5.09, 13.53) [19]. Compared with younger adults, the elderly (over 60 years old) are usually in poorer health and might be more vulnerable to PM_2.5_ and its chemical components exposure [42,43]. However, considering the sample size of patients over 60 years was relatively smaller (accounting for 10.57%), the association could be underestimated or overestimated when this factor is merely considered in the model as an extreme value.

There are some strengths of this study. Firstly, we provided very rare evidence of the associations of the short-term specific PM_2.5_ chemical component exposure with depression outpatient visits. Secondly, the time-stratified case-crossover design mitigated the connections of various confounding factors, such as socioeconomic status, education level, etc. The DLNM provided a more comprehensive understanding of the nonlinear and delayed influence of PM_2.5_ and its chemical components. Thirdly, in comparison to the data from space monitoring stations, the TAP dataset is time-sensitive and high-resolution, providing more accurate exposure information. Nevertheless, the study has some limitations. Our assessment of patients’ PM_2.5_ and its chemical components exposure was based on the resident address, which was unilateral that the patients were active within the area most of the time. In addition, there are certain potential confounding factors, such as green space and exercise frequency, which may affect the onset of depression. Moreover, this study only observed a lagged effect of 21 days, which might not fully capture the potential short-term effects of PM_2.5_ and its chemical components exposure on depression outpatient visits. Furthermore, we focused our research on only five significant chemical components of PM_2.5_, whereas other components, such as PAHs, Cu, Cd, Ni, and Zn, were not recorded because of the limitations of exposure data. Finally, this study did not explore the possible synergistic associations of the PM_2.5_ chemical components with the onset of depression, which needs to be compensated for in the future through better statistical models. 

## 5. Conclusions

Our findings add new evidence that short-term exposure to multiple specific components of PM_2.5_ might be an important risk factor for the depression incidence. Particular attention should be paid to SO_4_^2−^, OM, and their emission sources. We recommend that further regulations should be established focusing on the PM_2.5_ components. 

## Figures and Tables

**Table 1:** Daily outpatients for depression in three study cities during the study period.

Characteristics	Huizhou	Shenzhen	Zhaoqing	All
Gender				
Male	13 (47.92%)	74 (42.28%)	11 (41.45%)	98 (43.17%)
Female	14 (52.08%)	100 (57.72%)	15 (58.55%)	129 (56.83%)
Age				
<60 years	24 (87.93%)	158 (90.80%)	21 (82.28%)	203 (89.43%)
≥60 years	3 (12.07%)	16 (9.20%)	5 (17.72%)	24 (10.57%)

**Table 2:** Distributions of the PM_2.5_ chemical components and meteorological factors during the case and control days.

	Case Day	Control Day
No. of days	247,281	931,355
Meteorological factors		
Daily temperature (°C)	21.94 ± 5.63	22.03 ± 5.56
Relative humidity (%)	78.49 ± 12.11	78.63 ± 12.08
PM_2.5_ and its chemical components		
PM_2.5_ (μg/m^3^)	29.03 ± 15.00	28.96 ± 15.07
BC (μg/m^3^)	1.71 ± 0.94	1.71 ± 0.94
OM (μg/m^3^)	8.11 ± 4.50	8.10 ± 4.52
SO_4_^2−^ (μg/m^3^)	6.00 ± 3.29	6.00 ± 3.31
NO_3_^−^ (μg/m^3^)	3.86 ± 3.29	3.83 ± 3.28
NH_4_^+^ (μg/m^3^)	3.00 ± 2.32	2.98 ± 2.31

Notes: PM_2.5_ = fine particulate matter having an aerodynamic diameter of 2.5 μm or less; SO_4_^2−^ = sulfate; NO_3_^−^ = nitrate; NH_4_^+^ = ammonium; OM = organic matter; BC = black carbon.

**Table 3:** The cumulative risks of PM_2.5_ and its chemical components for outpatient visits for depression with *df* of 4, on lag 0–7, 0–14, 0–21 days, with the concentration that corresponds to the minimum effects serving as a reference.

Concentration (μg/m^3^)		Odds Ratio (95% CI)		
		Lag 0–7	Lag 0–14	Lag 0–21
PM_2.5_				
25th	18.1	1.207 (1.086, 1.341)	1.528 (1.305, 1.790)	1.525 (1.231, 1.889)
50th	25.9	1.181 (1.072, 1.302)	1.541 (1.332, 1.783)	1.607 (1.321, 1.956)
75th	36.5	1.127 (1.025, 1.239)	1.374 (1.193, 1.583)	1.403 (1.160, 1.697)
BC				
25th	1.0	0.941 (0.873, 1.013)	0.787 (0.704, 0.879)	0.684 (0.589, 0.795)
50th	1.5	0.941 (0.873, 1.013)	0.787 (0.704, 0.879)	0.684 (0.589, 0.795)
75th	2.2	0.962 (0.917, 1.009)	0.849 (0.791, 0.912)	0.766 (0.697, 0.843)
OM				
25th	4.7	1.036 (0.969, 1.107)	1.181 (1.069, 1.305)	1.231 (1.076, 1.408)
50th	7.2	1.044 (0.978, 1.114)	1.279 (1.161, 1.409)	1.417 (1.245, 1.612)
75th	10.4	1.007 (0.946, 1.072)	1.172 (1.069, 1.286)	1.277 (1.130, 1.443)
SO_4_^2−^				
25th	3.5	1.025 (1.006, 1.045)	1.077 (1.046, 1.109)	1.098 (1.057, 1.142)
50th	5.4	1.067 (1.016, 1.121)	1.218 (1.129, 1.313)	1.287 (1.164, 1.422)
75th	7.8	1.090 (1.023, 1.161)	1.302 (1.182, 1.435)	1.418 (1.247, 1.613)
NO_3_^−^				
25th	1.7	0.617 (0.551, 0.690)	0.459 (0.385, 0.547)	0.285 (0.226, 0.361)
50th	2.8	0.632 (0.567, 0.703)	0.487 (0.411, 0.577)	0.314 (0.250, 0.393)
75th	4.7	0.655 (0.588, 0.729)	0.539 (0.455, 0.638)	0.361 (0.288, 0.452)
NH_4_^+^				
25th	1.5	0.942 (0.905, 0.981)	0.867 (0.815, 0.921)	0.820 (0.755, 0.891)
50th	2.3	0.990 (0.983, 0.996)	0.975 (0.965, 0.985)	0.966 (0.953, 0.979)
75th	3.6	1.025 (1.009, 1.040)	1.065 (1.041, 1.090)	1.086 (1.054, 1.120)

Notes: The study adopted a DLNM approach that incorporates cross-basis functions. The concentrations were divided into the 25th percentile, 50th percentile, and 75th percentile to determine their effects. The reference levels of PM_2.5_, BC, OM, SO_4_^2−^, NO_3_^−^, NH_4_^+^ on lag 0–7 days were 1 μg/m^3^, 1.8 μg/m^3^, 9 μg/m^3^, 0.5 μg/m^3^, 0.5 μg/m^3^, 0.5 μg/m^3^, respectively. The reference levels of PM_2.5_, BC, OM, SO_4_^2−^, NO_3_^−^, NH_4_^+^ on lag 0–14 days were 1 μg/m^3^, 0.2 μg/m^3^, 1 μg/m^3^, 0.5 μg/m^3^, 10.9 μg/m^3^, 0.5 μg/m^3^, respectively. The reference levels of PM_2.5_, BC, OM, SO_4_^2−^, NO_3_^−^, NH_4_^+^ on lag 0–21 days were 1 μg/m^3^, 0.2 μg/m^3^, 1 μg/m^3^, 0.5 μg/m^3^, 8.9 μg/m^3^, 0.5 μg/m^3^, respectively.

## Data Availability

Publicly available datasets were analyzed in this study. This data can be found here: 10.6084/m9.figshare.24916242 (accessed on 29 December 2023).

## Supplementary Materials

The following supporting information can be downloaded at https://www.mdpi.com/article/10.3390/toxics12020136/s1, Figure S1: Box plots of exposure concentration of PM_2.5_ chemical components and the distribution density of the total study population at corresponding concentrations; Figure S2: Overall exposure-response relationships of PM_2.5_ and its chemical components with depression outpatient visits in the total study population at a 21-day lag in *df* of 4. The solid smooth lines and shaded areas represent the odds ratio of cause-specific mental disorder morbidity and its 95% CI, respectively. The horizontal dashed line in each panel indicates the odds ratio of 1; Figure S3: Overall exposure-response relationships of PM_2.5_ and its chemical components with depression outpatient visits at lag 21-day in each three cities. The solid smooth lines and shaded areas represent the odds ratio of cause-specific mental disorder morbidity and its 95% CI, respectively. The horizontal dashed line in each panel indicates the odds ratio of 1; Figure S4: Overall exposure-response relationships of PM_2.5_ and its chemical components with depression outpatient visits at lag 14-day in the total study population in *df* of 4. The solid smooth lines and shaded areas represent the odds ratio of cause-specific mental disorder morbidity and its 95% CI, respectively. The horizontal dashed line in each panel indicates the odds ratio of 1; Figure S5: Gender-stratified analysis for the cumulative association on lag 0–7, 0–14, 0–21 days at 50th percentile concentration of PM_2.5_ and its chemical components with depression outpatient visits; Figure S6: Age-stratified analyses for the cumulative association on lag 0–7, 0–14, 0–21 days at 50th percentile concentration of PM_2.5_ and its chemical components with depression outpatient visits; Figure S7: Overall exposure-response relationships of PM_2.5_ and its chemical components with depression outpatient visits at lag 21-day in the total study population. The solid smooth lines and shaded areas represent the odds ratio of cause-specific mental disorder morbidity and its 95% CI, respectively. The horizontal dashed line in each panel indicates the odds ratio of 1. (*df* = 3 for daily temperature and relative humidity); Figure S8: Overall exposure-response relationships of PM_2.5_ and its chemical components with depression outpatient visits at lag 21-day in the total study population. The solid smooth lines and shaded areas represent the odds ratio of cause-specific mental disorder morbidity and its 95% CI, respectively. The horizontal dashed line in each panel indicates the odds ratio of 1. (*df* = 5 for daily temperature and relative humidity); Table S1: Cross-validation of AICs of various *df* daily average air pollution; Table S2: Descriptive summary of the demographic characteristics of the three cities; Table S3: Spearman correlation among PM_2.5_ and its chemical components and meteorologic variables; Table S4: The cumulative effects of PM_2.5_ and its chemical components on depression outpatient visits with *df* of 4, on lag 0–7, 0–14, 0–21 days, at the 50th percentile concentration in each three cities, with the concentration corresponding to the minimum risk as the reference; Table S5: Gender stratified analysis for the cumulative effect on lag 0–7, 0–14, 0–21 days at 50th percentile concentration of PM_2.5_ and its chemical components with depression outpatient visits; Table S6: Age stratified analysis for the cumulative effect on lag 0–7, 0–14, 0–21 days at 50th percentile concentration of PM_2.5_ and its chemical components with depression outpatient visits; Table S7: The cumulative effects of PM_2.5_ and its chemical components on depression outpatient visits, on lag 0–7, 0–14, 0–21 days, with the concentration corresponding to the minimum risk as the reference. (*df* = 3 for daily temperature and relative humidity); Table S8: The cumulative effects of PM_2.5_ and its chemical components on depression outpatient visits, on lag 0–7, 0–14, 0–21 days, with the concentration corresponding to the minimum risk as the reference. (*df* = 5 for daily temperature and relative humidity).

## Abbreviations

PM_2.5_, fine particulate matter; DLNM, distributed lag nonlinear model; BC, black carbon; OM, organic matter; SO_4_^2−^, sulfate; NO_3_^−^, nitrate; NH_4_^+^, ammonium; OR, odd ratio; WHO, World Health Organization; HR, hazard ratio; ER, excess risk; IQR, interquartile range; ICD-10, International Classification of Disease-10; TAP, Tracking Air Pollution in China; ECMWF, European Centre for Medium-Range Weather Forecasts; SD, standard derivation; CI, confidence interval; *df*, degree of freedom; DSM-5, Diagnostic and Statistical Manual of Mental Disorders-5th Edition.
