# Supplementary material for: Short-Term Exposure to PM2.5 Chemical Components and Depression Outpatient Visits: A Case-Crossover Analysis in Three Chinese Cities
Source: Toxics. 2024 Feb 7;12(2):136. doi: 10.3390/toxics12020136 (PMC10892610; doi:10.3390/toxics12020136)
Supplement: Supplementary file 1 [file toxics-12-00136-s001.zip › toxics-2737866-supplementary.pdf]

Short-Term Exposure to $PM_{2.5}$ Chemical Components and Depression Outpatient Visits: A Case-Crossover Analysis in Three Chinese Cities

Zitong Zhuang [1,†], Dan Li [1,†], Shiyu Zhang [1], Zhaoyang Hu [1], Wenfeng Deng [2] and Hualiang Lin [1,*]

[1] School of Public Health, Sun Yat-Sen University, No. 74 Zhongshan Road 2, Guangzhou 510080, China

[2] Huizhou Center for Disease Control and Prevention, No. 10 Jiangbei Fumin Road, Huizhou 516003, China

* Correspondence: linhualiang@mail.sysu.edu.cn; Tel.: +86-020-87332455

† These authors contributed equally to this work.

Supplementary Materials

Table s1 Cross-validation of AICs of various *df* daily average air pollution

	AIC
air pollution (*df* = 2)	796773.0
air pollution (*df* = 3)	796658.5
air pollution (*df* = 4)	796566.1

Notes: AIC = Akaike information criterion; df = degree of freedom

Table s2. Descriptive summary of the demographic characteristics of the three cities.

	Huizhou		Shenzhen		Zhaoqing	
	Case day	Control day	Case day	Control day	Case day	Control day
N of days	58,112	197,424	189,156	642,238	27,013	91,693
Meteorologic variables						
Daily temperature (℃)	22.12±5.77	22.21±5.69	21.87±5.56	21.96±5.50	22.08±5.78	22.20±5.66
Relative humidity (%)	78.24±12.95	78.43±12.96	78.44±12.02	78.55±12.01	79.33±10.82	79.63±10.46
Concentrations of $PM_{2.5}$ and its chemical composition						
$PM_{2.5}$ (μg/m^3)	29.86±15.90	29.80±15.98	28.73±14.20	28.67±14.34	29.32±18.06	29.21±17.71
BC (μg/m^3)	1.87±1.04	1.87±1.04	1.68±0.91	1.68±0.91	1.55±0.86	1.55±0.84
OM (μg/m^3)	8.28±4.66	8.25±4.65	8.15±4.44	8.14±4.48	7.48±4.45	7.46±4.41
SO_4^{2-} (μg/m^3)	6.36±3.31	6.28±3.34	5.90±3.24	5.89±3.28	5.90±3.50	5.89±3.41
NO_3^- (μg/m^3)	4.52±3.57	4.45±3.61	3.42±2.74	3.37±2.73	5.50±4.99	5.45±4.90
NH_4^+ (μg/m^3)	3.73±2.72	3.68±2.74	2.61±1.82	2.57±1.82	4.14±3.43	4.12±3.35

Notes: $PM_{2.5}$ = fine particulate matter having an aerodynamic diameter of 2.5 μm or less; SO_4^{2-} = sulfate; NO_3^- = nitrate; NH_4^+ = ammonium; OM = organic matter; BC = black carbon.

Table s3. Spearman correlation among $PM_{2.5}$ and its chemical components and meteorologic variables.

	$PM_{2.5}$	SO_4^{2-}	NO_3^-	NH_4^+	OM	BC	Daily temperature	Relative humidity
$PM_{2.5}$	1	—	—	—	—	—	—	—
SO_4^{2-}	0.96	1	—	—	—	—	—	—
NO_3^-	0.87	0.84	1	—	—	—	—	—
NH_4^+	0.87	0.84	0.97	1	—	—	—	—
OM	0.97	0.95	0.81	0.80	1	—	—	—
BC	0.93	0.94	0.76	0.77	0.96	1	—	—
Daily temperature	-0.32	-0.30	-0.55	-0.50	-0.29	-0.24	1	—
Relative humidity	-0.36	-0.32	-0.49	-0.44	-0.36	-0.28	0.53	1

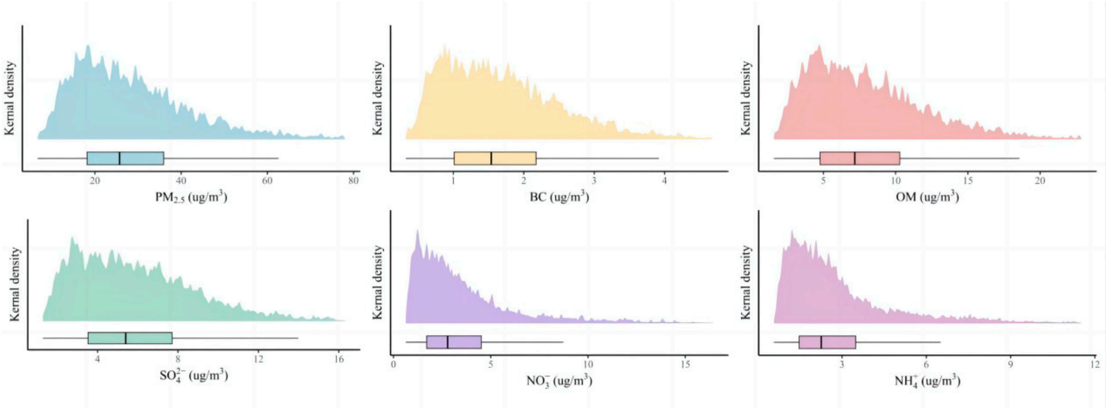

Figure s1. Box plots of exposure concentration of $PM_{2.5}$ chemical components and the distribution density of the total study population at corresponding concentrations.

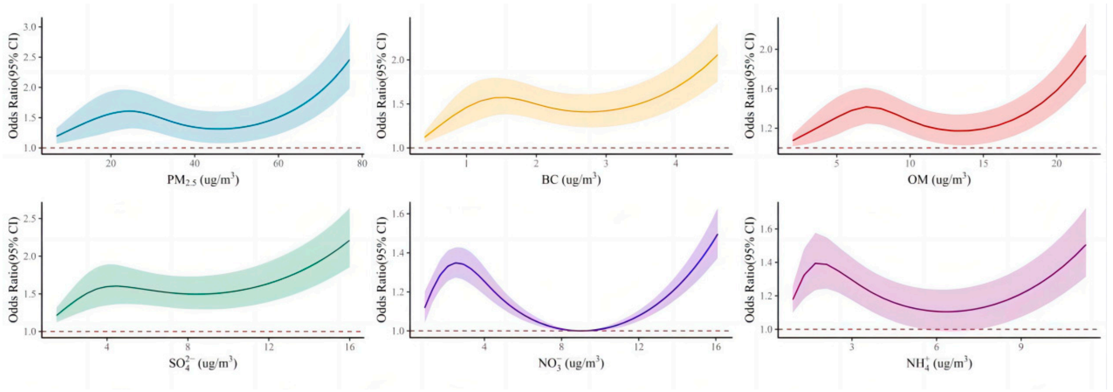

Figure s2. Overall exposure-response relationships of $PM_{2.5}$ and its chemical components with depression outpatient visits in the total study population at a 21-day lag in *df* of 4. The solid smooth lines and shaded areas represent the odds ratio of cause-specific mental disorder morbidity and its 95% CI, respectively. The horizontal dashed line in each panel indicates the odds ratio of 1.

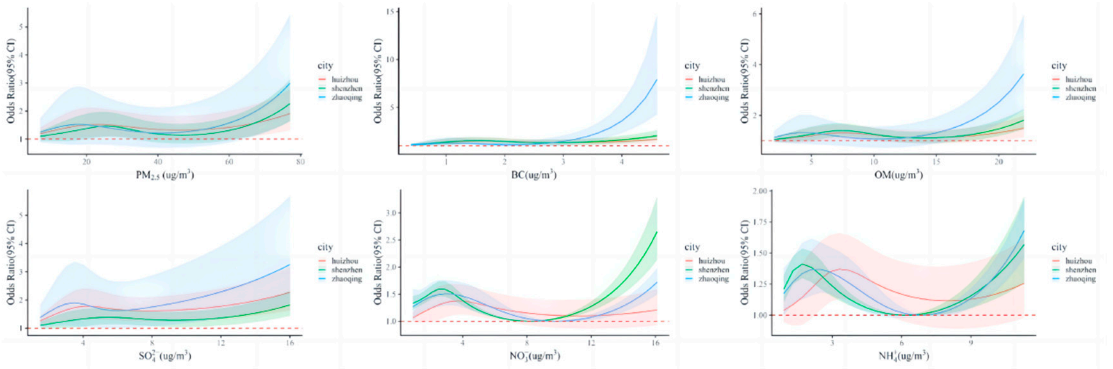

Figure s3. Overall exposure-response relationships of $PM_{2.5}$ and its chemical components with depression outpatient visits at lag 21-day in each three cities. The solid smooth lines and shaded areas represent the odds ratio of cause-specific mental disorder morbidity and its 95% CI, respectively. The horizontal dashed line in each panel indicates the odds ratio of 1.

Table s4. The cumulative effects of $PM_{2.5}$ and its chemical components on depression outpatient visits with *df* of 4, on lag 0-7, 0-14, 0-21 days, at the 50th percentile concentration in each three cities, with the concentration corresponding to the minimum risk as the reference.

Concentration(μg/m^3)		Odds Ratio (95% CI)		
		Lag 0-7	Lag 0-14	Lag 0-21
Huizhou				
$PM_{2.5}$	26.1	1.132 (0.959, 1.336)	1.436 (1.131, 1.823)	1.510 (1.104, 2.065)
BC	1.7	0.962 (0.847, 1.094)	0.831 (0.692, 0.998)	0.734 (0.578, 0.933)
OM	7.2	1.042 (0.925, 1.174)	1.246 (1.052, 1.474)	1.317 (1.056, 1.644)
SO_4^{2-}	5.7	1.100 (1.008, 1.201)	1.263 (1.109, 1.439)	1.359 (1.148, 1.610)
NO_3^-	3.4	1.008 (0.982, 1.035)	1.027 (0.988, 1.068)	1.045 (0.993, 1.099)
NH_4^+	2.9	0.998 (0.987, 1.009)	0.992 (0.976, 1.008)	0.990 (0.969, 1.011)
Shenzhen				
$PM_{2.5}$	26.0	1.169 (1.016, 1.346)	1.525 (1.230, 1.891)	1.465 (1.091, 1.967)
BC	1.5	0.934 (0.833, 1.046)	0.775 (0.649, 0.925)	0.713 (0.559, 0.908)
OM	7.4	1.019 (0.930, 1.117)	1.276 (1.107, 1.470)	1.405 (1.162, 1.699)
SO_4^{2-}	5.4	1.015 (0.950, 1.085)	1.140 (1.028, 1.264)	1.146 (0.998, 1.317)
NO_3^-	2.6	0.616 (0.510, 0.744)	0.373 (0.281, 0.494)	0.174 (0.116, 0.260)
NH_4^+	2.1	0.961 (0.756, 1.221)	0.645 (0.456, 0.912)	0.363 (0.220, 0.598)
Zhaoqing				
$PM_{2.5}$	24.0	1.060 (0.781, 1.438)	1.234 (0.795, 1.915)	1.446 (0.809, 2.584)

BC	1.3	1.126 (0.914, 1.386)	0.919 (0.691, 1.221)	0.791 (0.543, 1.154)
OM	6.3	0.981 (0.798, 1.205)	1.074 (0.801, 1.440)	1.259 (0.849, 1.866)
SO_4^{2-}	5.0	1.102 (0.927, 1.312)	1.271 (0.984, 1.640)	1.476 (1.055, 2.066)
NO_3^-	3.6	0.412 (0.332, 0.513)	0.233 (0.162, 0.335)	0.108 (0.066, 0.178)
NH_4^+	3	0.428 (0.340, 0.537)	0.238 (0.165, 0.343)	0.111 (0.067, 0.184)

Table s5. Gender stratified analysis for the cumulative effect on lag 0-7, 0-14, 0-21 days at 50th percentile concentration of $PM_{2.5}$ and its chemical components with depression outpatient visits.

	Odds Ratio (95% CI)					
	Lag 0-7		Lag 0-14		Lag 0-21	
	Male	Female	Male	Female	Male	Female
$PM_{2.5}$	1.121 (0.969, 1.297)	1.232 (1.081, 1.404)	1.399 (1.124, 1.740)	1.667 (1.371, 2.028)	1.377 (1.027, 1.847)	1.820 (1.398, 2.369)
BC	0.946 (0.846, 1.059)	0.934 (0.846, 1.033)	0.836 (0.707, 0.988)	0.748 (0.645, 0.868)	0.749 (0.598, 0.938)	0.635 (0.520, 0.777)
OM	1.004 (0.910, 1.107)	1.077 (0.987, 1.175)	1.178 (1.018, 1.363)	1.367 (1.201, 1.556)	1.255 (1.034, 1.524)	1.561 (1.314, 1.855)
SO_4^{2-}	1.054 (0.980, 1.135)	1.078 (1.009, 1.151)	1.175 (1.049, 1.316)	1.253 (1.133, 1.386)	1.179 (1.014, 1.370)	1.380 (1.206, 1.578)
NO_3^-	0.679 (0.578, 0.797)	0.992 (0.985, 1.000)	0.536 (0.417, 0.689)	0.982 (0.970, 0.993)	0.361 (0.258, 0.505)	0.971 (0.956, 0.986)
NH_4^+	0.996 (0.986, 1.006)	0.985 (0.976, 0.994)	0.982 (0.967, 0.998)	0.969 (0.956, 0.983)	0.976 (0.956, 0.997)	0.958 (0.941, 0.976)

Table s6. Age stratified analysis for the cumulative effect on lag 0-7, 0-14, 0-21 days at 50th percentile concentration of $PM_{2.5}$ and its chemical components with depression outpatient visits.

	Odds Ratio (95% CI)					
	Lag 0-7		Lag 0-14		Lag 0-21	
	＜60	≥60	＜60	≥60	＜60	≥60
$PM_{2.5}$	1.168 (1.054, 1.295)	1.285 (0.962, 1.716)	1.523 (1.305, 1.778)	1.695 (1.103, 2.605)	1.559 (1.266, 1.920)	2.051 (1.154, 3.645)
BC	0.941 (0.869, 1.018)	0.935 (0.750, 1.166)	0.789 (0.701, 0.889)	0.762 (0.551, 1.055)	0.696 (0.593, 0.816)	0.601 (0.389, 0.929)
OM	1.035 (0.966, 1.109)	1.123 (0.924, 1.365)	1.262 (1.139, 1.399)	1.435 (1.075, 1.915)	1.379 (1.202, 1.581)	1.765 (1.203, 2.590)
SO_4^{2-}	1.063 (1.010, 1.120)	1.099 (0.946, 1.275)	1.218 (1.124, 1.319)	1.220 (0.971, 1.533)	1.286 (1.156, 1.430)	1.297 (0.958, 1.757)
NO_3^-	0.657 (0.585, 0.738)	0.995 (0.978, 1.012)	0.513 (0.428, 0.616)	0.986 (0.961, 1.012)	0.342 (0.268, 0.437)	0.973 (0.941, 1.006)
NH_4^+	0.990 (0.983, 0.997)	0.988 (0.969, 1.009)	0.975 (0.965, 0.986)	0.973 (0.943, 1.003)	0.968 (0.954, 0.982)	0.953 (0.915, 0.992)

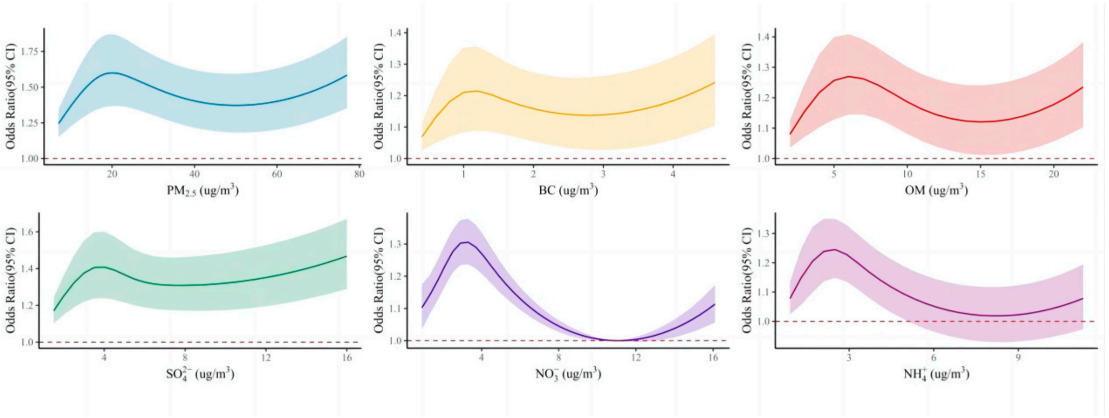

Figure s4. Overall exposure-response relationships of $PM_{2.5}$ and its chemical components with depression outpatient visits at lag 14-day in the total study population in *df* of 4. The solid smooth lines and shaded areas represent the odds ratio of cause-specific mental disorder morbidity and its 95% CI, respectively. The horizontal dashed line in each panel indicates the odds ratio of 1.

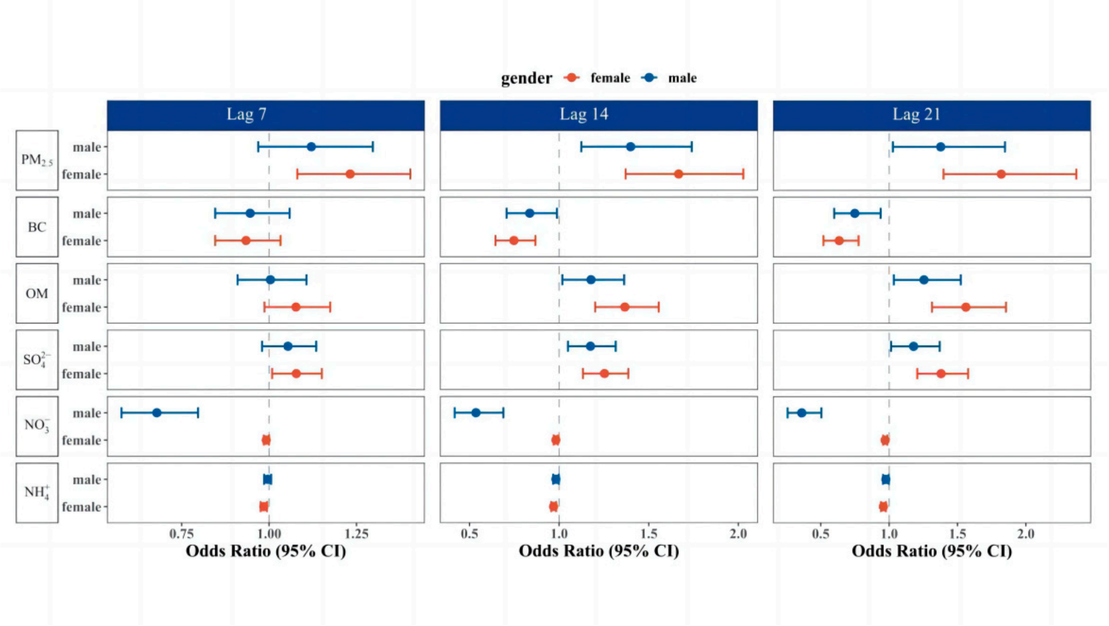

Figure s5. Gender-stratified analysis for the cumulative association on lag 0-7, 0-14, 0-21 days at 50th percentile concentration of $PM_{2.5}$ and its chemical components with depression outpatient visits.

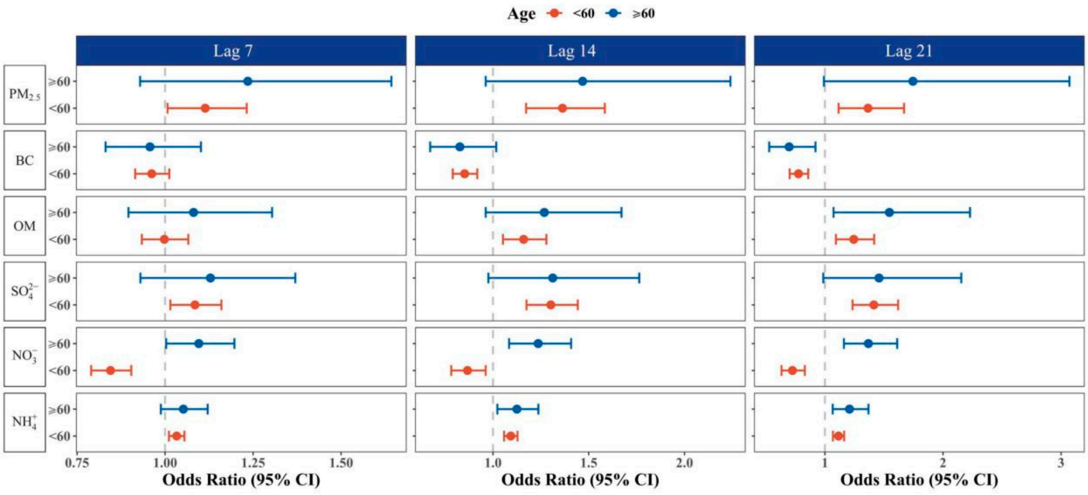

Figure s6. Age-stratified analyses for the cumulative association on lag 0-7, 0-14, 0-21 days at 50th percentile concentration of $PM_{2.5}$ and its chemical components with depression outpatient visits.

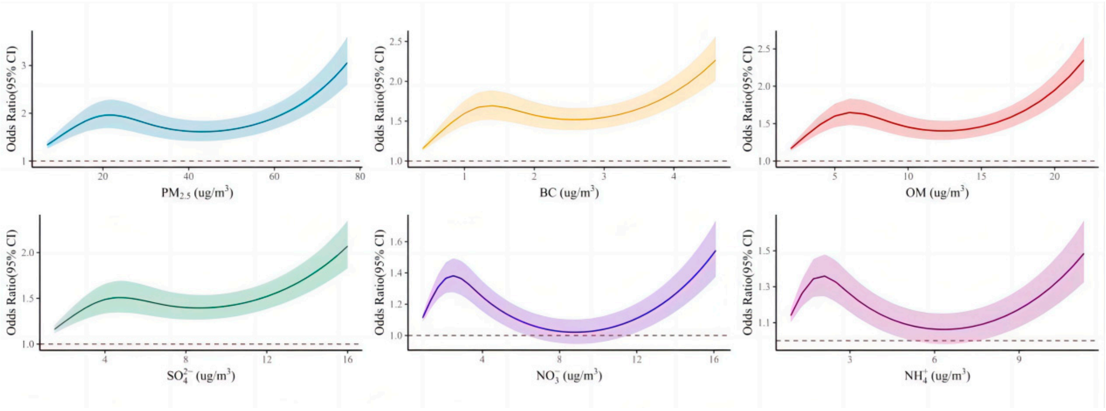

Figure s7. Overall exposure-response relationships of $PM_{2.5}$ and its chemical components with depression outpatient visits at lag 21-day in the total study population. The solid smooth lines and shaded areas represent the odds ratio of cause-specific mental disorder morbidity and its 95% CI, respectively. The horizontal dashed line in

each panel indicates the odds ratio of 1. (*df* = 3 for daily temperature and relative humidity)

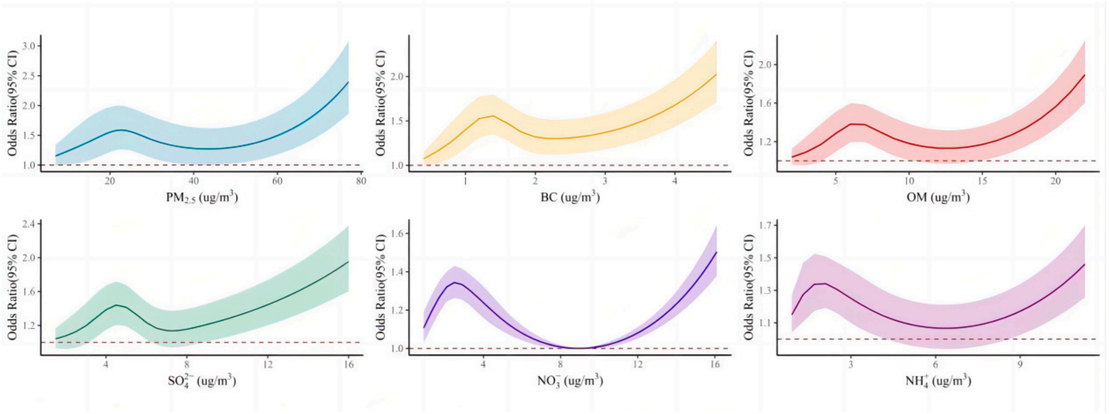

Figure s8. Overall exposure-response relationships of $PM_{2.5}$ and its chemical components with depression outpatient visits at lag 21-day in the total study population. The solid smooth lines and shaded areas represent the odds ratio of cause-specific mental disorder morbidity and its 95% CI, respectively. The horizontal dashed line in each panel indicates the odds ratio of 1. (*df* = 5 for daily temperature and relative humidity)

Table s7. The cumulative effects of $PM_{2.5}$ and its chemical components on depression outpatient visits, on lag 0-7, 0-14, 0-21 days, with the concentration corresponding to the minimum risk as the reference. (*df* = 3 for daily temperature and relative humidity)

Concentration ($\mu g/m^3$)		Odds Ratio (95% CI)		
		Lag 0-7	Lag 0-14	Lag 0-21
$PM_{2.5}$				
25th	18.1	1.189 (1.107, 1.277)	1.615 (1.451, 1.798)	1.909 (1.658, 2.197)
50th	25.9	1.182 (1.093, 1.278)	1.616 (1.438, 1.815)	1.924 (1.652, 2.241)
75th	36.5	1.122 (1.044, 1.206)	1.427 (1.283, 1.587)	1.664 (1.449, 1.911)
BC				
25th	1.0	0.947 (0.901, 0.995)	0.780 (0.724, 0.839)	0.625 (0.568, 0.688)
50th	1.5	0.947 (0.901, 0.995)	0.780 (0.724, 0.839)	0.625 (0.568, 0.688)
75th	2.2	0.964 (0.931, 0.998)	0.843 (0.801, 0.887)	0.723 (0.676, 0.773)
OM				
25th	4.7	1.079 (1.035, 1.125)	1.306 (1.226, 1.391)	1.488 (1.371, 1.615)
50th	7.2	1.084 (1.025, 1.147)	1.374 (1.264, 1.494)	1.629 (1.463, 1.815)
75th	10.4	1.045 (0.993, 1.100)	1.250 (1.160, 1.348)	1.452 (1.318, 1.599)
SO_4^{2-}				
25th	3.5	1.016 (1.005, 1.026)	1.046 (1.030, 1.062)	1.076 (1.055, 1.098)
50th	5.4	1.043 (1.014, 1.073)	1.130 (1.083, 1.179)	1.224 (1.159, 1.293)
75th	7.8	1.061 (1.018, 1.106)	1.191 (1.119, 1.268)	1.342 (1.238, 1.454)
NO_3^-				

25th	1.7	0.959 (0.945, 0.974)	0.897 (0.877, 0.917)	0.877 (0.852, 0.902)
50th	2.8	0.992 (0.989, 0.995)	0.980 (0.976, 0.984)	0.976 (0.970, 0.981)
75th	4.7	1.045 (1.027, 1.063)	1.123 (1.095, 1.151)	1.148 (1.112, 1.185)
NH_4^+				
25th	1.5	0.953 (0.933, 0.974)	0.879 (0.851, 0.907)	0.852 (0.818, 0.887)
50th	2.3	0.991 (0.987, 0.995)	0.977 (0.971, 0.982)	0.971 (0.964, 0.979)
75th	3.6	1.023 (1.012, 1.033)	0.977 (0.971, 0.982)	1.077 (1.056, 1.098)

Table s8. The cumulative effects of $PM_{2.5}$ and its chemical components on depression outpatient visits, on lag 0-7, 0-14, 0-21 days, with the concentration corresponding to the minimum risk as the reference. (*df* = 5 for daily temperature and relative humidity)

Concentration ($\mu g/m^3$)		Odds Ratio (95% CI) Lag 0-7	Lag 0-14	Lag 0-21
$PM_{2.5}$				
25th	18.1	1.160 (1.023, 1.314)	1.552 (1.282, 1.879)	1.493 (1.159, 1.922)
50th	25.9	1.148 (1.026, 1.285)	1.554 (1.309, 1.844)	1.561 (1.244, 1.959)
75th	36.5	1.061 (0.938, 1.200)	1.352 (1.121, 1.630)	1.315 (1.027, 1.683)
BC				
25th	1.0	0.969 (0.892, 1.052)	0.790 (0.698, 0.894)	0.714 (0.606, 0.840)
50th	1.5	0.969 (0.892, 1.052)	0.790 (0.698, 0.894)	0.714 (0.606, 0.840)
75th	2.2	0.964 (0.918, 1.012)	0.842 (0.783, 0.906)	0.766 (0.696, 0.843)
OM				

25th	4.7	1.020 (0.939, 1.108)	1.173 (1.034, 1.330)	1.172 (0.993, 1.384)
50th	7.2	1.044 (0.973, 1.120)	1.276 (1.146, 1.420)	1.376 (1.195, 1.585)
75th	10.4	0.985 (0.909, 1.068)	1.132 (1.004, 1.278)	1.180 (1.007, 1.383)
SO_4^{2-}				
25th	3.5	0.997 (0.971, 1.023)	1.026 (0.985, 1.069)	1.024 (0.971, 1.079)
50th	5.4	1.006 (0.945, 1.071)	1.096 (0.995, 1.208)	1.106 (0.974, 1.256)
75th	7.8	1.047 (0.977, 1.123)	1.205 (1.081, 1.343)	1.267 (1.100, 1.460)
NO_3^-				
25th	1.7	0.639 (0.564, 0.724)	0.432 (0.352, 0.531)	0.285 (0.217, 0.373)
50th	2.8	0.634 (0.567, 0.708)	0.466 (0.389, 0.560)	0.309 (0.243, 0.393)
75th	4.7	0.651 (0.583, 0.727)	0.519 (0.434, 0.620)	0.354 (0.280, 0.447)
NH_4^+				
25th	1.5	0.940 (0.889, 0.994)	0.846 (0.776, 0.922)	0.844 (0.752, 0.947)
50th	2.3	0.990 (0.982, 0.998)	0.973 (0.961, 0.985)	0.971 (0.954, 0.987)
75th	3.6	1.021 (1.006, 1.037)	1.059 (1.035, 1.084)	1.074 (1.042, 1.107)
